# Structured headache services as the solution to the ill-health burden of headache. 2. Modelling effectiveness and cost-effectiveness of implementation in Europe: methodology

**DOI:** 10.1186/s10194-021-01310-x

**Published:** 2021-08-23

**Authors:** Michela Tinelli, Matilde Leonardi, Koen Paemeleire, Dimos Mitsikostas, Elena Ruiz de la Torre, Timothy J. Steiner, on behalf of the European Brain Council Value of Treatment Headache Working Group, the European Headache Federation, the European Federation of Neurological Associations, and Lifting The Burden: the Global Campaign against Headache

**Affiliations:** 1 Care Policy Evaluation Centre, The London School of Economics and Political Science, Houghton Street, London, WC2A 2AE UK; 2 Fondazione IRCCS Istituto Neurologico Carlo Besta, Milan, Italy; 3 Department of Neurology, Ghent University Hospital, Ghent, Belgium; 4 1st Department of Neurology, Aeginition Hospital, Medical School, National and Kapodistrian University of Athens, Athens, Greece; 5 European Migraine and Headache Alliance, Brussels, Belgium; 6 Department of Neuromedicine and Movement Science, Norwegian University of Science and Technology, Trondheim, Norway; 7 Division of Brain Sciences, Imperial College London, London, UK

**Keywords:** Headache, Migraine, Tension-type-headache (TTH), Medication-overuse-headache (MOH), Structured headache services, Health economics, Cost-effectiveness, Quality improvement, Healthy-life-years (HLYs), Global campaign against headache

## Abstract

**Background:**

Health economic evaluations support health-care decision-making by providing information on the costs and consequences of health interventions. No universally accepted methodology exists for modelling effectiveness and cost-effectiveness of interventions designed to close treatment gaps for headache disorders in countries of Europe (or elsewhere). Our aim here, within the European Brain Council’s Value-of-Treatment project, was to develop headache-type-specific analytical models to be applied to implementation of structured headache services in Europe as the health-care solution to headache.

**Methods:**

We developed three headache-type-specific decision-analytical models using the WHO-CHOICE framework and adapted these for three European Region country settings (Luxembourg, Russia and Spain), diverse in geographical location, population size, income level and health-care systems and for which we had population-based data. Each model compared current (suboptimal) care vs target care (delivered in accordance with the structured headache services model). Epidemiological and economic data were drawn from studies conducted by the Global Campaign against Headache; data on efficacy of treatments were taken from published randomized controlled trials; assumptions on uptake of treatments, and those made for Healthy Life Year (HLY) calculations and target-care benefits, were agreed with experts. We made annual and 5-year cost estimates from health-care provider (main analyses) and societal (secondary analyses) perspectives (2020 figures, euros).

**Results:**

The analytical models were successfully developed and applied to each country setting. Headache-related costs (including use of health-care resources and lost productivity) and health outcomes (HLYs) were mapped across populations. The same calculations were repeated for each alternative (current vs target care). Analyses of the differences in costs and health outcomes between alternatives and the incremental cost-effectiveness ratios are presented elsewhere.

**Conclusions:**

This study presents the first headache-type-specific analytical models to evaluate effectiveness and cost-effectiveness of implementing structured headache services in countries in the European Region. The models are robust, and can assist policy makers in allocating health budgets between interventions to maximize the health of populations.

## Background

Headache disorders, principally migraine, tension-type headache (TTH) and medication-overuse headache (MOH), are responsible for 5.4% of all disability in the world and were the cause in 2019 of an estimated 46.6 million years lived with disability (YLDs) globally [1, 2]. Most (88.2%) of these were attributable to migraine [3], recognized in successive iterations of the Global Burden of Disease (GBD) study as the world’s second leading cause of disability [1, 4–6]. Because disability leads to lost productivity, headache disorders have substantial financial impact. Each million of the population in Europe loses an estimated 400,000 days from work or school every year to migraine alone, while the estimated cost of headache disorders in Europe, due in the main to lost productivity, is well in excess of €100 billion per year [7].

**Fig. 1:**
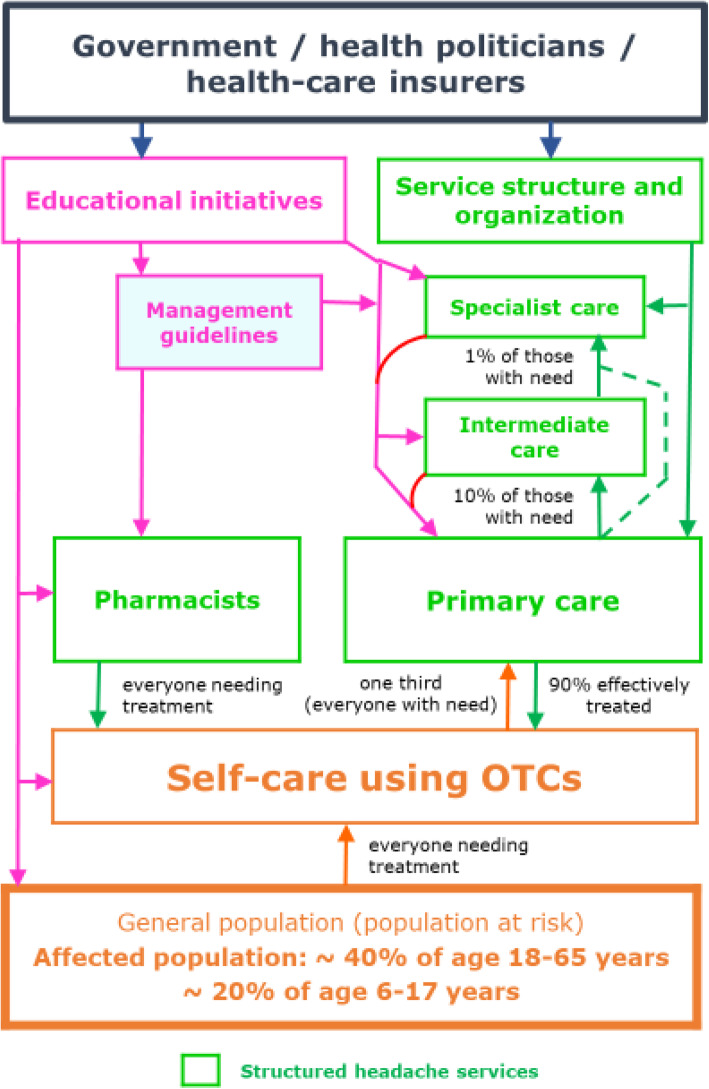
Template for structured headache services supported by educational initiatives, and expected patient flows (as described in [9]: structured headache services are based in primary care and supported by specialist care; educational initiatives are aimed at health-care providers to improve competence at their level, and at the public to promote self-care and effective use of both over-the-counter (OTC) drugs and headache services; pharmacists advise on use of OTC and other drugs, discouraging overuse, and on use of headache services; within these services, everyone with headache should make best use of OTC drugs; about one third of people with headache need professional health care; primary care provides effective management for most of these, while specialist care is reserved for the small proportion who need it)

Effective treatments exist for these disorders [8] but are under-utilized, largely because, in all countries, health-care systems fail to provide them [9]. The reasons are complex and not for discussion here, but they have their roots in health policies that trenchantly deny headache disorders the priority they clearly deserve [10] in view of the ill health they cause [1–6]. The solution – structured headache services (Fig. 1) – has been proposed [9], but its adoption will depend – rightly in a universal context of competition for resources – on economic evidence of cost-effectiveness, value and return on investment.

### EBC’s value-of-treatment project

In 2015, the European Brain Council (EBC) initiated its Value-of-Treatment (VoT) project, building on the success of its Cost of Brain Disorders database [11, 12]. VoT set out to identify obstacles, pinch-points and dead-ends in the “patient’s journey” through each of nine common mental and neurological diseases, including headache, then specify remedies and, in its ultimate purpose, assess the value of improvements made in line with these remedies [13].

In the headache case, the inefficiencies and failures of the care pathway are described in previous manuscripts [9, 14]. Structured headache services as the specific remedy are based in primary care in order to provide sufficient reach, with recourse to specialist services at second and third levels for the relatively few patients needing these [9]. Implementation requires educational supports at all levels, for the general public as well as health-care providers, which are built into the model [9]. With these supports, only about one third of people with headache should need professional care at any level, and primary care should effectively manage about 90% of these [9].

As for *value*, structured headache services reaching all who may benefit will be costly to implement. The up-front investment will be substantial, but so is the expected recovery of lost health [9]. Our aim here, through decision-analytical modelling, is to generate the required evidence of value needed to influence policy.

No universally accepted methodology exists for modelling effectiveness and cost-effectiveness of service-delivery interventions designed to close headache treatment gaps. This paper reports our development of methods and their use. We describe current care and the treatment management plan to achieve target care, the types of intervention, and the coverage and uptake estimates used in three headache-type-specific decision-analytical models. We explain how we calculate economic and health outcomes, and report the key results of applying the three analytical models to population data from three paradigmatic countries in the European Region, including healthy life years (HLYs) gained and cost differences when changing from current to target care. The full economic analyses are reported elsewhere [15].

## Methods

We selected three countries of the World Health Organization (WHO) European Region: Luxembourg, Russia and Spain, diverse in geographical location, population size, level of income and health-care systems. Important in these choices was that, for each, we had population-based data to support the analyses [16–18].

**Fig. 2:**
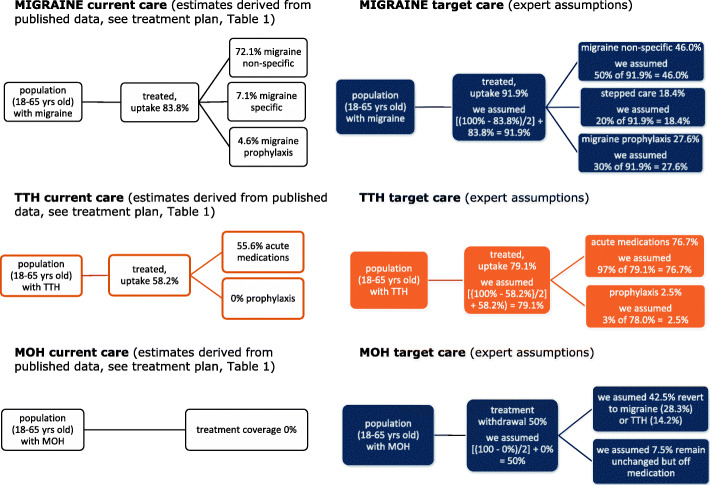
Analytical models for Luxembourg (data are reported in Table 1)

**Fig. 3:**
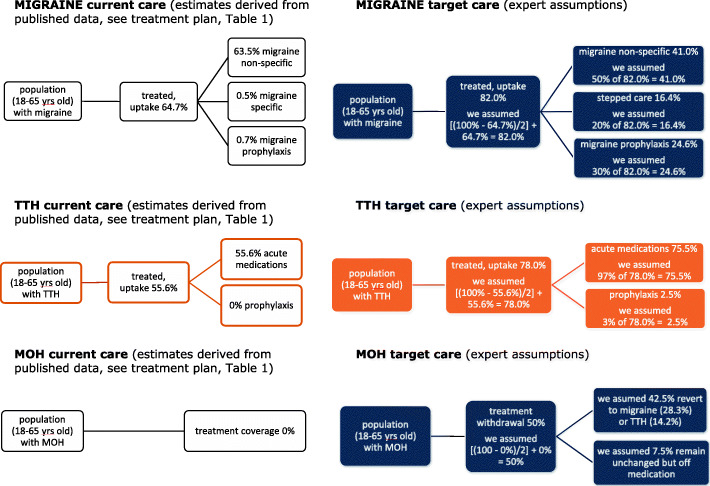
Analytical models for Russia (data are reported in Table 2)

**Fig. 4:**
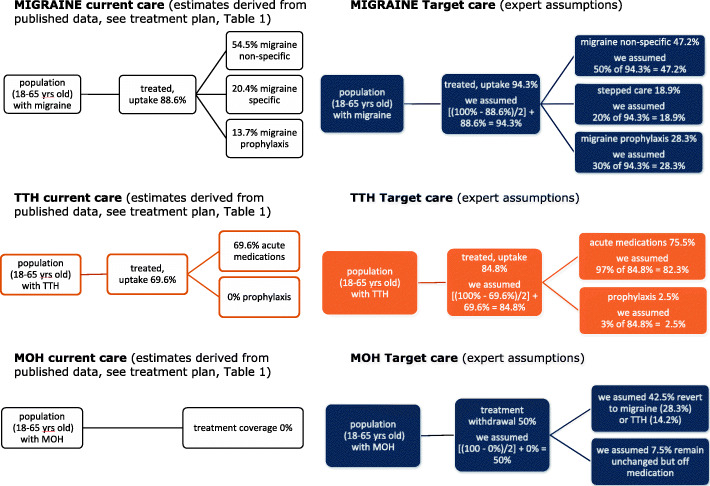
Analytical models for Spain (data are reported in Table 3)

We developed three separate headache-type-specific decision-analytical models from an earlier exercise using the WHO-CHOICE framework [19], and simulated outcomes for the populations of 18–65-year-olds with migraine, TTH or MOH. In each model, two alternatives were compared: current (suboptimal) care vs target care delivered in accordance with the structured headache services model (see Figs. 2, 3 and 4). We made annual and 5-year cost estimates from health-care provider (main analyses) and societal perspectives (secondary analyses) (2020 figures, euros). We expressed effectiveness as HLYs gained and cost-effectiveness as incremental cost-effectiveness ratios (ICERs) (cost to be invested/HLY gained). We applied WHO thresholds to establish cost-effectiveness: interventions costing <3x gross domestic product (GDP) per capita per HLY were deemed cost-effective, those costing <1x GDP per capita were highly cost-effective [25]. In comparisons of current vs target care, we made the assumptions that implemented structured services with provider-training would achieve higher coverage (the proportion of people in need of a treatment who receive it), and consumer-education would lead to better adherence (the proportion who use a treatment effectively, having received it), in each case, conservatively, by 50% of the gap between current and ideal. Economic outputs included direct costs (resources sunk into health-care provision) and indirect costs (lost work productivity [see secondary analyses]). We performed sensitivity analyses with regard to how much lost productivity might be recovered to test robustness of the model.

### Decision-analytical models*:* treatment management plan, selection of interventions and medicines uptake

We developed and applied the three headache-type-specific decision-analytical models to each country setting. For each alternative (current and target care), we adopted a core set of drug interventions, focusing on those included in Linde et al [19]. Among these were first-line (simple analgesics: *eg*, acetylsalicylic acid (ASA) 1000 mg) and second-line medications (*eg*, sumatriptan 50 mg) for acute treatment of attacks, with the assumption that the latter would be used only by non-responders to the former in a stepped-care treatment paradigm [8]. We also included preventative drugs (*eg*, amitriptyline 100 mg daily) to be used by those with ≥3.5 headache days/month. For target care, we added to the model the expected consequences of consumer education (posters and leaflets in pharmacies explaining how to acquire and make best use of these medications) and of health-care provider training.

The treatment plans for the three headache types are described in Tables 1, 2 and 3.

**Table 1:** Treatment uptake, use of resources and lost productivity according to treatment management plan (Luxembourg)

	Headache type	Current care (%)	Target care (%)	Notes	
Uptake (including coverage and adherence)	**Migraine**	83.8	91.9	**Current care**: 72.1% migraine non-specific; 7.1% migraine specific; 4.6% migraine prophylaxis (see treatment plan below) **Target care**: We assumed that structured services with consumer education and provider training enhances coverage and adherence so that uptake is increased by 50% of current deficit: medicines uptake = [{100% - 83.8%}/2] + 83.8%) = 91.9%	
	**TTH**	58.2	79.1	**Current care**: 58.2% acute medications; 0% TTH prophylaxis (see treatment plan below) **Target care**: We assumed as above: medicines uptake = [{100% - 58.2%}/2] + 58.2%) = 79.1%	
	**MOH**	0	50.0	**Current care**: 0% treated **Target care**: We assumed that structured services with consumer education and provider training enhances treatment coverage and adherence so that proportion withdrawn from medicines overuse is increased by 50% of current deficit: withdrawal = [{100–0%}/2] + 0%) = 50.0%	
TREATMENT PLAN					
A. Acute management (non-specific drugs)					
Simple analgesics (*eg*, ASA 1 g)		**Migraine**	72.1	46.0	**Current care**: from Eurolight data [20] **Target care:** With consumer education and provider training, treatment with simple analgesics alone is used by or offered to 50% (expert assumption), with uptake = 46.0% (50% of 91.9%)
		**TTH**	55.6	76.7	**Current care**: from Eurolight data [20] **Target care**: With consumer education and provider training, treatment with simple analgesics alone is used by or offered to 97% (expert assumption), with uptake 76.7% (97% of 79.1%)
		**MOH**	0	0	Not applicable to MOH care
B. Acute management (specific drugs)					
Sumatriptan 50 mg		**Migraine**	7.1	0	**Current care**: from Eurolight data [20] **Target care**: With provider training, treatment with specific drugs alone is offered to 0% (expert assumption)
		**TTH**	0	0	Not applicable to TTH care
		**MOH**	0	0	Not applicable to MOH care
C. Acute stepped-care management					
ASA 1 g + sumatriptan 50 mg		**Migraine**	0	18.4	**Current care**: not included in current care **Target care**: With provider training, acute stepped-care management is offered to 20% (expert assumption), with uptake = 18.4% (20% of 91.9%)
		**TTH**	0	0	Not applicable to TTH care
		**MOH**	0	0	Not applicable to MOH care
D. Prophylaxis + acute management					
Amitriptyline 100 mg/day + ASA 1 g + sumatriptan 50 mg		**Migraine**	4.6	27.6	**Current care**: from Eurolight data [20] **Target care**: With provider training, prophylaxis + acute stepped-care management is offered to 30% (expert assumption), with uptake = 27.6% (30% of 91.9%)
		**TTH**	0	2.4	**Current care**: not included in current care **Target care**: With provider training, prophylaxis + acute care management is offered to 3% (expert assumption), with uptake = 2.4% (3% of 79.1%)
		**MOH**	0	0	Not applicable to MOH care
Consultations and investigations					
Doctor visits (year 1)		**Migraine**	25.1	50.0	**Current care**: 25.1% with migraine had seen a doctor (Eurolight data [20]), of whom 19.3% had seen a GP and 5.8% a specialist. We assumed 2 visits in either case. **Target care**: With consumer education, 50% see a doctor (expert assumption based on estimated need for professional care). Note that in the model those who see a specialist would see a GP first.
		**TTH**	9.4	2.25	**Current care**: 9.4% with TTH had seen a doctor (Eurolight data [20]), of whom 6.9% had seen a GP and 2.5% a specialist. We assumed 2 visits in either case. **Target care**: With consumer education, 3% (Stovner 2007 [21]) × 75% = 2.25% see a specialist and none see a GP (expert assumption based on estimated need for professional care). Note that those who see a specialist would see a GP first.
		**MOH**	51.2	100	**Current care**: 51.2% with MOH had seen a doctor (Eurolight data [20]), of whom 21.6% had seen a GP and 29.6% a specialist. We assumed 2 visits in either case. **Target care**: With consumer education, 100% see a doctor (expert assumption based on estimated need for professional care). Note that those who see a specialist would see a GP first.
GP visits		**Migraine**	19.3	45.0	**Current care**: 19.3% had seen a GP (Eurolight data [20]) **Target care**: With consumer education, 45.0% (90% of 50%) see a GP (we assumed 2 visits in a year)
		**TTH**	6.9	0	**Current care**: 6.9% had seen a GP (2 times in a year) (Eurolight data [20]) **Target care**: Chronic TTH is difficult to treat, so we assumed that all should go to levels 2 or 3 (ie, “specialists”). Note that those who see a specialist would see a GP first.
		**MOH**	21.6	100	**Current care**: 21.6% had seen a GP (2 times in a year) (Eurolight data [20]) **Target care**: With consumer education, 100% see a GP (we assumed 2 visits in a year)
Specialist visits		**Migraine**	5.8	5.0	**Current care**: 5.8% had seen a specialist (2 times in a year) **Target care**: With consumer education and provider training, 5.0% (10% of 50%) see a specialist (we assumed 2 visits in a year)
		**TTH**	2.5	2.25	**Current care**: 2.5% had seen a specialist (2 times in a year) **Target care**: With consumer education and provider training, 2.25% see a specialist (we assumed 2 visits in a year)
		**MOH**	29.6	100	**Current care:** 29.6% saw a GP (2 times in a year) **Target care:** With consumer education and provider training, 100% see a specialist (we assumed 2 visits in a year)
Investigations (MRI) (year 1)		**Migraine**	8.5	1.0	**Current care**: All those seeing a specialist had MRI (one in a year) **Target care**: With provider training, we assumed 1% have MRI (one in a year)
		**TTH**	1.0	0.5	**Current care**: 1% had an MRI **Target care**: We assumed 0.5% have MRI examination (one in a year) – half the current estimate
		**MOH**	0	0	**Current care**: Nobody had an MRI **Target care**: Nobody has an MRI
Doctor visits (years 2–5)		**Migraine**	24.6	50.0	**Current care**: 24.6% with migraine had seen a doctor (Eurolight data [20]), of whom all saw a GP only after year 1. We assumed 2 visits per year. **Target care**: With consumer education, 50% see a doctor (expert assumption based on estimated need for professional care)
		**TTH**	9.4	2.25	**Current care**: 9.4% with TTH had seen a doctor (Eurolight data [20]), of whom all saw a GP only after year 1. We assumed 2 visits per year. **Target care:** With consumer education, 3% (Stovner 2007 [21]) × 75% = 2.25% see a doctor (expert assumption based on estimated need for professional care). Note that those who see a specialist would see a GP first.
		**MOH**	51.2	100	**Current care**: 51.2% with MOH had seen a doctor (Eurolight data [20]), of whom all saw a GP only after year 1. We assumed 2 visits per year. **Target care**: With consumer education, 100% see a doctor
GP visits		**Migraine**	24.6	50.0	**Current care**: 24.6% saw a GP. We assumed 2 visits each year. **Target care**: With consumer education, 50% see a GP. We assumed 2 visits each year.
		**TTH**	9.4	0	**Current care**: 9.4% saw a GP. We assumed 2 visits each year. **Target care**: Chronic TTH is difficult to treat, so we assumed that all should go to levels 2 or 3 (ie, “specialists”). Note that those who see a specialist would see a GP first.
		**MOH**	51.2	100	**Current care:** 51.2% saw a GP. We assumed 2 visits each year. **Target care**: With consumer education, 100% see a GP. We assumed 2 visits each year.
Specialist visits		**Migraine**	0	0	**Current care**: No visits after year 1 **Target care**: No visits after year 1
		**TTH**	0	2.25	**Current care**: No visits after year 1 **Target care**: With consumer education and provider training, 2.25% see a specialist (we assumed 2 visits in a year).
		**MOH**	0	0	**Current care**: No visits after year 1 **Target care**: No visits after year 1
Investigation (MRI) (years 2–5)		**Migraine**	0	0	**Current care**: nobody had an MRI after year 1 **Target care**: nobody had an MRI after year 1
		**TTH**	0	0	**Current care**: nobody had an MRI after year 1 **Target care**: nobody had an MRI after year 1
		**MOH**	0	0	**Current care**: nobody had an MRI after year 1 **Target care**: nobody had an MRI after year 1
Lost productivity			We assumed that lost work productivity was correlated with disease-attributed disability, and reduced disability would bring reduced lost productivity. In our baseline scenario, all lost productivity was explained by disease-attributed disability.		
Days lost from work in 12 months		**Migraine**	7.6	2.4	**Current care**: Based on Eurolight data [16] **Target care**: We assumed 69% decrease in lost productivity (equal to the gain in HLYs reported for migraine (see Table 4)): 7.6-(7.6*0.69) = 2.4 days.
		**TTH**	3.2	1.0	**Current care**: Based on Eurolight data [16] **Target care**: We assumed 76% decrease in lost productivity (equal to the gain in HLYs reported for TTH (see Table 4)): 3.2-(3.2*0.76) = 1.0 days.
		**MOH**	22.8	7.1 (if revert to migraine); 5.5 (if revert to TTH)	**Current care:** Based on Eurolight data [16] **Target care:** For individuals reverting to migraine, we assumed 69% decrease in lost productivity (equal to the gain in HLYs reported for migraine (see Table 4)): 22.8-(22.8*0.69) = 7.1 days For individuals reverting to TTH, we assumed 76% decrease in lost productivity (equal to the gain in HLYs reported for TTH (see Table 4)): 22.8-(22.8*0.76) = 5.5 days.

**Table 2:** Treatment uptake, use of resources and lost productivity according to treatment management plan (Russia)

	Headache type	Current care (%)	Target care (%)	Notes
Uptake (including coverage and adherence)	**Migraine**	64.7	82.0	**Current care:** 63.5% migraine non-specific; 0.5% migraine specific; 0.7% migraine prophylaxis **Target care:** We assumed that structured services with consumer education and provider training enhances coverage and adherence so that overall uptake is increased by 50%; medicines uptake = [{100% - 64.7%}/2] + 64.7%) = 82.0%
	**TTH**	55.6	77.8	55.6% (acute medications) TTH; 0% TTH prophylaxis (see treatment plan below) **Target care:** We assumed as above; medicines uptake = [{100% - 55.6%}/2] + 55.6%) = 77.8%
	**MOH**	0	50.0	**Current care**: 0% treated **Target care:** We assumed that structured services with consumer education and provider training enhances treatment coverage and adherence so that proportion withdrawn from medicines overuse is increased by 50% of current deficit: withdrawal = [{100–0%}/2] + 0%) = 50%
TREATMENT PLAN				
A. Acute management (non-specific drugs)				
Simple analgesics (*eg*, ASA 1 g)	**Migraine**	63.5	41.0	**Current care**: from Eurolight data [20] **Target care:** With provider training, treatment with simple analgesics alone is offered to 50% (expert assumption), with uptake = 41.0% (50% of 82.0%)
	**TTH**	55.6	75.5	**Current care**: from Eurolight data [20] **Target care**: With provider training, treatment with simple analgesics alone is offered to 97% (expert assumption), with uptake = 75.5% (97% of 77.8%)
	**MOH**	0	0	Not applicable to MOH care
B. Acute management (specific drugs)				
Sumatriptan 50 mg	**Migraine**	0.5	0	**Current care**: from Eurolight data [20] **Target care**: With provider training, treatment with specific drugs alone is offered to 0% (expert assumption)
	**TTH**	0	0	Not applicable to TTH care
	**MOH**	0	0	Not applicable to MOH care
C. Acute stepped-care management				
ASA 1 g + sumatriptan 50 mg	**Migraine**	0	16.4	**Current care**: not included in current care **Target care**: With provider training, acute stepped-care management is offered to 20% (expert assumption), with uptake = 16.4% (20% of 82.0%)
	**TTH**	0	0	Not applicable to TTH care
	**MOH**	0	0	Not applicable to MOH care
D. Prophylaxis + acute management				
Amitriptyline 100 mg/day + ASA 1 g + sumatriptan 50 mg	**Migraine**	0.7	24.6	**Current care**: from Eurolight data [20] **Target care**: With provider training, prophylaxis + acute stepped-care management is offered to 30% (expert assumption), with uptake = 24.6% (30% of 82.0%)
	**TTH**	0	2.3	**Current care**: not included in current care **Target care**: With provider training, prophylaxis + acute care management is offered to 3% (expert assumption), with uptake = 2.3% (3% of 77.8%)
	**MOH**	0	0	Not applicable to MOH care
Consultations and investigations				
Doctor visits (year 1)	**Migraine**	25.1	50.0	**Current care**: 25.1% with migraine had seen a doctor (Eurolight data [20]), of whom 19.3% had seen a GP and 5.8% a specialist. We assumed 2 visits in either case. **Target care**: With consumer education, 50% see a doctor (expert assumption based on estimated need for professional care). Note that in the model those who see a specialist would see a GP first.
	**TTH**	9.4	2.25	**Current care**: 9.4% with TTH had seen a doctor (Eurolight data [20]), of whom 6.9% had seen a GP and 2.5% a specialist. We assumed 2 visits in either case. **Target care**: With consumer education, 3% (Stovner 2007 [21]) × 75% = 2.25% see a specialist and none see a GP (expert assumption based on estimated need for professional care). Note that those who see a specialist would see a GP first.
	**MOH**	51.2	100	**Current care**: 51.2% with MOH had seen a doctor (Eurolight data [20]), of whom 21.6% had seen a GP and 29.6% a specialist. We assumed 2 visits in either case. **Target care**: With consumer education, 100% see a doctor (expert assumption based on estimated need for professional care). Note that those who see a specialist would see a GP first.
GP visits	**Migraine**	19.3	45.0	**Current care**: 19.3% had seen a GP (Eurolight data [20]) **Target care**: With consumer education, 45.0% (90% of 50%) see a GP (we assumed 2 visits in a year)
	**TTH**	6.9	0	**Current care**: 6.9% had seen a GP (2 times in a year) (Eurolight data [20]) **Target care**: Chronic TTH is difficult to treat, so we assumed that all should go to levels 2 or 3 (ie, “specialists”). Note that those who see a specialist would see a GP first.
	**MOH**	21.6	100	**Current care**: 21.6% had seen a GP (2 times in a year) (Eurolight data [20]) **Target care**: With consumer education, 100% see a GP (we assumed 2 visits in a year)
Specialist visits	**Migraine**	5.8	5.0	**Current care**: 5.8% had seen a specialist (2 times in a year) **Target care**: With consumer education and provider training, 5.0% (10% of 50%) see a specialist (we assumed 2 visits in a year)
	**TTH**	2.5	2.25	**Current care**: 2.5% had seen a specialist (2 times in a year) **Target care**: With consumer education and provider training, 2.25% see a specialist (we assumed 2 visits in a year)
	**MOH**	29.6	100	**Current care:** 29.6% saw a GP (2 times in a year) **Target care:** With consumer education and provider training, 100% see a specialist (we assumed 2 visits in a year)
Investigations (MRI) (year one)	**Migraine**	8.5	1.0	**Current care**: All those seeing a specialist had MRI (one in a year) **Target care**: With provider training, we assumed 1% have MRI (one in a year)
	**TTH**	1.0	0.5	**Current care**: 1% had an MRI **Target care**: We assumed 0.5% have MRI examination (one in a year) – half the current estimate
	**MOH**	0	0	**Current care**: Nobody had an MRI **Target care**: Nobody has an MRI
Doctor visits (years 2–5)	**Migraine**	24.6	50.0	**Current care**: 24.6% with migraine had seen a doctor (Eurolight data [20]), of whom all saw a GP only after year 1. We assumed 2 visits per year. **Target care**: With consumer education, 50% see a doctor (expert assumption based on estimated need for professional care)
	**TTH**	9.4	2.25	**Current care**: 9.4% with TTH had seen a doctor (Eurolight data [20]), of whom all saw a GP only after year 1. We assumed 2 visits per year. **Target care:** With consumer education, 3% (Stovner 2007 [21]) × 75% = 2.25% see a doctor (expert assumption based on estimated need for professional care). Note that those who see a specialist would see a GP first.
	**MOH**	51.2	100	**Current care**: 51.2% with MOH had seen a doctor (Eurolight data [20]), of whom all saw a GP only after year 1. We assumed 2 visits per year. **Target care**: With consumer education, 100% see a doctor
GP visits	**Migraine**	24.6	50.0	**Current care**: 24.6% saw a GP. We assumed 2 visits each year. **Target care**: With consumer education, 50% see a GP. We assumed 2 visits each year.
	**TTH**	9.4	0	**Current care**: 9.4% saw a GP. We assumed 2 visits each year. **Target care**: Chronic TTH is difficult to treat, so we assumed that all should go to levels 2 or 3 (ie, “specialists”). Note that those who see a specialist would see a GP first.
	**MOH**	51.2	100	**Current care:** 51.2% saw a GP. We assumed 2 visits each year. **Target care**: With consumer education, 100% see a GP. We assumed 2 visits each year.
Specialist visits	**Migraine**	0	0	**Current care**: No visits after year 1 **Target care**: No visits after year 1
	**TTH**	0	2.25	**Current care**: No visits after year 1 **Target care**: With consumer education and provider training, 2.25% see a specialist (we assumed 2 visits in a year).
	**MOH**	0	0	**Current care**: No visits after year 1 **Target care**: No visits after year 1
Investigation (MRI) (years 2–5)	**Migraine**	0	0	**Current care**: nobody had an MRI after year 1 **Target care**: nobody had an MRI after year 1
	**TTH**	0	0	**Current care**: nobody had an MRI after year 1 **Target care**: nobody had an MRI after year 1
	**MOH**	0	0	**Current care**: nobody had an MRI after year 1 **Target care**: nobody had an MRI after year 1
Lost productivity	We assumed that lost work productivity is correlated with disease-related disability, and reduced disability would bring reduced lost productivity. In our baseline scenario, all lost productivity was explained by disease-related disability.			
Days lost from work in 12 months	**Migraine**	7.6	3.9	**Current care**: based on Eurolight data [16] **Target care**: we assumed 49% decrease in lost productivity (equal to the gain in HLYs reported for migraine (see Table 4)): 7.6-(7.6*0.49) = 3.9 days.
	**TTH**	3.2	1.0	**Current care**: based on Eurolight data [16] **Target care**: we assumed 68% decrease in lost productivity (equal to the gain in HLYs reported for TTH (see Table 4)): 3.2-(3.2*0.68) = 1.0 days.
	**MOH**	22.8	11.6 (if revert to migraine); 7.3 (if revert to TTH)	**Current care:** based on Eurolight data [16] **Target care:** for individuals reverting to migraine, we assumed 49% decrease in lost productivity (equal to the gain in HLYs reported for migraine (see Table 4)): 22.8 - (22.8*0.49) = 11.6 days for individuals reverting to TTH, we assumed 76% decrease in lost productivity (equal to the gain in HLYs reported for TTH (see Table 4)): 22.8-(22.8*0.68) = 7.3 days.

**Table 3:** Treatment uptake, use of resources and lost productivity according to treatment management plan (Spain)

	Headache type	Current care (%)	Target care (%)	Notes	
Uptake (including coverage and adherence)	**Migraine**	88.6	94.3	**Current care**: 54.5% migraine non-specific + 20.4% migraine specific + 13.7% migraine prophylaxis = 88.6% (see treatment plan below) **Target care**: We assumed that structured services with consumer education and provider training enhances coverage and adherence so that uptake is increased by 50% of current deficit: medicines uptake = [{100% - 88.6%}/2] + 88.6%) = 94.3%	
	**TTH**	69.6	84.8	**Current care**: 69.6% acute medications; 0% TTH prophylaxis (see treatment plan below) **Target care**: We assumed as above: medicines uptake [{100% - 69.6%}/2] + 69.6%) = 84.8%	
	**MOH**	0	50.0	**Current care**: 0% treated **Target care**: We assumed that structured services with consumer education and provider training enhances treatment coverage and adherence so that proportion withdrawn from medicines overuse is increased by 50% of current deficit: withdrawal = [{100–0%}/2] + 0%) = 50.0%	
TREATMENT PLAN					
A. Acute management (non-specific drugs)					
Simple analgesics (*eg*, ASA 1 g)		**Migraine**	54.5	47.2	**Current care**: from Eurolight data [20] **Target care:** With provider training, treatment with simple analgesics alone is offered to 50% (expert assumption), with uptake = 47.2% (50% of 94.3%)
		**TTH**	69.6	82.3	**Current care**: from Eurolight data [20] **Target care**: With provider training, treatment with simple analgesics alone is offered to 97% (expert assumption), with uptake = 82.3% (97% of 84.8%)
		**MOH**	0	0	Not applicable to MOH care
B. Acute management (specific drugs)					
Sumatriptan 50 mg		**Migraine**	20.4	0	**Current care**: from Eurolight data [20] **Target care**: With provider training, treatment with specific drugs alone is offered to 0% (expert assumption)
		**TTH**	0	0	Not applicable to TTH care
		**MOH**	0	0	Not applicable to MOH care
C. Acute stepped care management					
ASA 1 g + sumatriptan 50 mg		**Migraine**	0	18.9	**Current care**: not included in current care **Target care**: With provider training, acute stepped-care management is offered to 20% (expert assumption), with uptake = 18.9% (20% of 94.3%)
		**TTH**	0	0	Not applicable to TTH care
		**MOH**	0	0	Not applicable to MOH care
D. Prophylaxis + acute management					
Amitriptyline 100 mg/day + ASA 1 g + sumatriptan 50 mg		**Migraine**	13.7	28.3	**Current care**: from Eurolight data [20] **Target care**: With provider training, prophylaxis + acute stepped-care management is offered to 30% (expert assumption), with uptake = 28.3% (30% of 94.3%)
		**TTH**	0	2.5	**Current care**: not included in current care **Target care**: With provider training, prophylaxis + acute care management is offered to 3% (expert assumption), with uptake = 2.5% (3% of 84.8%)
		**MOH**	0	0	Not applicable to MOH care
Consultations and investigations					
Doctor visits (year 1)		**Migraine**	25.1	50.0	**Current care**: 25.1% with migraine had seen a doctor (Eurolight data [20]), of whom 19.3% had seen a GP and 5.8% a specialist. We assumed 2 visits in either case. **Target care**: With consumer education, 50% see a doctor (expert assumption based on estimated need for professional care). Note that in the model those who see a specialist would see a GP first.
		**TTH**	9.4	2.25	**Current care**: 9.4% with TTH had seen a doctor (Eurolight data [20]), of whom 6.9% had seen a GP and 2.5% a specialist. We assumed 2 visits in either case. **Target care**: With consumer education, 3% (Stovner 2007 [21]) × 75% = 2.25% see a specialist and none see a GP (expert assumption based on estimated need for professional care). Note that those who see a specialist would see a GP first.
		**MOH**	51.2	100	**Current care**: 51.2% with MOH had seen a doctor (Eurolight data [20]), of whom 21.6% had seen a GP and 29.6% a specialist. We assumed 2 visits in either case. **Target care**: With consumer education, 100% see a doctor (expert assumption based on estimated need for professional care). Note that those who see a specialist would see a GP first.
GP visits		**Migraine**	19.3	45.0	**Current care**: 19.3% had seen a GP (Eurolight data [20]) **Target care**: With consumer education, 45.0% (90% of 50%) see a GP (we assumed 2 visits in a year)
		**TTH**	6.9	0	**Current care**: 6.9% had seen a GP (2 times in a year) (Eurolight data [20]) **Target care**: Chronic TTH is difficult to treat, so we assumed that all should go to levels 2 or 3 (ie, “specialists”). Note that those who see a specialist would see a GP first.
		**MOH**	21.6	100	**Current care**: 21.6% had seen a GP (2 times in a year) (Eurolight data [20]) **Target care**: With consumer education, 100% see a GP (we assumed 2 visits in a year)
Specialist visits		**Migraine**	5.8	5.0	**Current care**: 5.8% had seen a specialist (2 times in a year) **Target care**: With consumer education and provider training, 5.0% (10% of 50%) see a specialist (we assumed 2 visits in a year)
		**TTH**	2.5	2.25	**Current care**: 2.5% had seen a specialist (2 times in a year) **Target care**: With consumer education and provider training, 2.25% see a specialist (we assumed 2 visits in a year)
		**MOH**	29.6	100	**Current care:** 29.6% saw a GP (2 times in a year) **Target care:** With consumer education and provider training, 100% see a specialist (we assumed 2 visits in a year)
Investigations (MRI) (year one)		**Migraine**	8.5	1.0	**Current care**: All those seeing a specialist had MRI (one in a year) **Target care**: With provider training, we assumed 1% have MRI (one in a year)
		**TTH**	1.0	0.5	**Current care**: 1% had an MRI **Target care**: We assumed 0.5% have MRI examination (one in a year) – half the current estimate
		**MOH**	0	0	**Current care**: Nobody had an MRI **Target care**: Nobody has an MRI
Doctor visits (years 2–5)		**Migraine**	24.6	50.0	**Current care**: 24.6% with migraine had seen a doctor (Eurolight data [20]), of whom all saw a GP only after year 1. We assumed 2 visits per year. **Target care**: With consumer education, 50% see a doctor (expert assumption based on estimated need for professional care)
		**TTH**	9.4	2.25	**Current care**: 9.4% with TTH had seen a doctor (Eurolight data [20]), of whom all saw a GP only after year 1. We assumed 2 visits per year. **Target care:** With consumer education, 3% (Stovner 2007 [21]) × 75% = 2.25% see a doctor (expert assumption based on estimated need for professional care). Note that those who see a specialist would see a GP first.
		**MOH**	51.2	100	**Current care**: 51.2% with MOH had seen a doctor (Eurolight data [20]), of whom all saw a GP only after year 1. We assumed 2 visits per year. **Target care**: With consumer education, 100% see a doctor
GP visits		**Migraine**	24.6	50.0	**Current care**: 24.6% saw a GP. We assumed 2 visits each year. **Target care**: With consumer education, 50% see a GP. We assumed 2 visits each year.
		**TTH**	9.4	0	**Current care**: 9.4% saw a GP. We assumed 2 visits each year. **Target care**: Chronic TTH is difficult to treat, so we assumed that all should go to levels 2 or 3 (ie, “specialists”). Note that those who see a specialist would see a GP first.
		**MOH**	51.2	100	**Current care:** 51.2% saw a GP. We assumed 2 visits each year. **Target care**: With consumer education, 100% see a GP. We assumed 2 visits each year.
Specialist visits		**Migraine**	0	0	**Current care**: No visits after year 1 **Target care**: No visits after year 1
		**TTH**	0	2.25	**Current care**: No visits after year 1 **Target care**: With consumer education and provider training, 2.25% see a specialist (we assumed 2 visits in a year).
		**MOH**	0	0	**Current care**: No visits after year 1 **Target care**: No visits after year 1
Investigation (MRI) (years 2–5)		**Migraine**	0	0	**Current care**: nobody had an MRI after year 1 **Target care**: nobody had an MRI after year 1
		**TTH**	0	0	**Current care**: nobody had an MRI after year 1 **Target care**: nobody had an MRI after year 1
		**MOH**	0	0	**Current care**: nobody had an MRI after year 1 **Target care**: nobody had an MRI after year 1
Lost productivity		We assumed that lost work productivity is correlated with disease-related disability, and reduced disability would bring reduced lost productivity. In our baseline scenario, all lost productivity was explained by disease-related disability.			
Days lost from work in 12 months		**Migraine**	7.6	2.4	**Current care**: based on Eurolight data [16] **Target care**: we assumed 69% decrease in lost productivity (equal to the gain in HLYs reported for migraine (see Table 4)): 7.6-(7.6*0.69) = 2.4 days
		**TTH**	3.2	0.8	**Current care**: based on Eurolight data [16] **Target care**: we assumed 76% decrease in lost productivity (equal to the gain in HLYs reported for TTH (see Table 4)): 3.2-(3.2*0.76) = 0.8 days
		**MOH**	22.8	7.1 (if revert to migraine); 5.5 (if revert to TTH)	**Current care:** based on Eurolight data [16] **Target care:** for individuals reverting to migraine, we assumed 69% decrease in lost productivity (equal to the gain in HLYs reported for migraine (see Table 4)): 22.8-(22.8*0.69) = 7.1 days for individuals reverting to TTH, we assumed 76% decrease in lost productivity (equal to the gain in HLYs reported for TTH (see Table 4)): 22.8-(22.8*0.76) = 5.5 days

We estimated uptake (%) of each treatment in current care (U_cc_) in each of the three settings according to coverage and adherence. We took coverage data from Global Campaign studies [17, 18], including the Eurolight project [16, 20], and followed Linde et al [19] on adherence (see Tables 1, 2, and 3). For target care, we calculated predicted uptake (U_tc_) as {[(100 – U_cc_)/2] + U_cc_}%. All details on the data used and assumptions made to calculate uptake are in Tables 1, 2 and 3. We adjusted estimates of efficacy from published clinicaltrials by reference to uptake, better to reflect effectiveness in the real world.

### Economic outcomes: use of resources and lost productivity according to treatment management plan

Use of resources and lost productivity data were taken or extrapolated from different sources (Tables 1, 2 and 3) [7, 8, 20, 26]. Unit costs for health-care resources (medicines, consultations, examinations) [19] and daily wages [27] are reported in Table 12 in Appendix. We actualised costs in euros to 2020 values using the appropriate consumer price index [28]. At population level, the relationship between lost productivity and headache-attributed disability is complex (people are variably influenced by a number of extraneous and sometimes random factors) (Hallie Thomas, Simple Futarmal Kothari, Andreas Husøy, Rigmor Hølland Jensen, Zaza Katsarava, Michela Tinell and Timothy J Steiner. The relationship between headache-attributed disability and lost productivity. 2. Empirical evidence from population-based studies in six disparate countries forthcoming). We therefore performed sensitivity analyses with regard to this. In our baseline sensitivity scenario, all lost productivity was explained by headache-attributed disability, whereas, in an alternative scenario, measurable disability accounted for only 20% of lost productivity.

### Health outcomes: epidemiological data, disability, and estimation of intervention effectiveness

We ran a population model for the two alternatives (current vs target care) over one- and five-year time frames to estimate total HLYs lived by the populations in each country in each alternative. The differences between these two simulations represented the population-level health gain (HLYs gained) from the intervention relative to current care. Epidemiological data were sourced from Global Campaign surveys performed in the three countries [1] (see Table 4).

**Table 4:** Epidemiological data, disability weights, prevalence, frequency and duration of attacks

		Luxembourg	Russia	Spain		
		Value	Value	Value	Source	Specification
Population, overall		586,869	143,500,000	46,064,604	[22]	2016 values
Proportion 18–65 yrs. old, %		70	70.6	66	[23]	
DW (% ictal disability)	migraine	44.1	44.1	44.1	[24]	2015 values
	TTH	3.7	3.7	3.7	[24]	2015 values
	MOH	21.7	21.7	21.7	[24]	2015 values
Prevalence %	migraine	30.358	17.888	35.432	[1]	
	TTH	31.037	26.334	25.821	[1]	
	MOH	3.500	7.1	7.000	[1]	
Mean frequency days/month	migraine	4.4/30	4.4/30	4.4/30	[24]	
	TTH	3.5/30	3.5/30	3.5/30	[24]	
	MOH	23.1/30	23.1/30	23.1/30	[24]	
Mean duration, hours (current)	migraine	15	15	15	[24]	
	TTH	7.4	7.4	7.4	[24]	
Mean duration, hours (target)	migraine	2	2	2	expert opinion	
	TTH	2	2	2	expert opinion	
YLDs (current)	migraine	0.00040	0.00024	0.00047	[24]	
	TTH	0.00005	0.00003	0.00006	[24]	
	MOH	0.00020	0.00017	0.00017	[24]	
YLDs (target)	migraine	0.00005	0.00005	0.00005		
	TTH	0.00585	0.01186	0.01170		

*DW* disability weight; TTH: tension type headache; MOH: medication-overuse headache; YLDs: years of healthy life lost to disability. YLDs = product of prevalence, mean frequency, mean duration and DW

We applied separate disability weights (DWs) (health state valuations on a 0–1 scale, where 1 equals full health) to the times spent in the ictal state (within-attack) and interictal state (between attacks, but susceptible). Ictal DWs (0.441 for migraine, 0.037 for TTH and 0.217 for MOH) were available from GBD2015 ([24]; Table 4). For interictal DW in each disorder, to reflect interictal disability [29], we used the lowest possible weighting of 0.01 and applied it only to those with high-frequency attacks (> 3.5/month). For migraine and TTH, we calculated headache-attributed disability at individual level in YLDs as the product of proportion of time in ictal state (pTIS: itself estimated as a product of attack frequency (F) and mean duration), with and without intervention, and the DW for the disorder in question. For MOH, we assumed pTIS was equal to (days/month affected)/30. To estimate disability at population level, we multiplied the means of these values by prevalence of the respective disorder.

Epidemiological data [16–18], including attack frequencies and durations [19], and DWs [24] for the different models are summarised in Table 4.

We modelled treatment effect as reduction in pTIS, adopting the universal outcome measure previously developed for this purpose [30] but, since this was a population-level analysis, expressing effect in terms of HLYs gained rather than hours lived with disability (HLDs) averted. Accordingly, for acute medicines, we used the clinical endpoint of “sustained headache relief” (SHR), defined as reduction in headache intensity from moderate or severe to mild or none within 2 h and without recurrence or further medication during 24 h. We assumed baseline headache was always at least moderate, and that mild and no pain were not associated with disability. SHR therefore implied full recovery of the remaining hours of the attack that would have been spent with disability [19, 30]. We assumed that treatment was taken at attack onset, so that hours recovered per treated attack = D-2, where D = expected attack duration in hours [19, 30]. Thus:

- pTIS_untreated_ = D * F
- pTIS_treated_ = [(D-2)/D * (pF_treated_ * pSHR)] + {D * [(1- (pF_treated_ * pSHR)]}
- reduction in pTIS = pTIS_untreated_ – pTIS_treated_

where: F = attack frequency; pF_treated_ = proportion treated; pSHR = efficacy expressed as proportion of treated attacks with SHR.

We assessed the effect of acute management and its combination in high-frequency cases with preventative drugs (modifying F), together with the potential effects of provider training on treatment coverage (modifying pF_treated_) and of consumer education on adherence (modifying pF_treated_ and pSHR). Data on efficacy (from randomized controlled trials) and uptake are listed in Table 5 [23, 31–35]. For MOH we assumed success in 85% of treated cases, with reversion to other types of headache (*ie*, 2/3 to migraine and 1/3 to TTH); the other 15% would remain unchanged, but off medications.

**Table 5:** Assumptions adopted when calculating the health effects

Efficacy	
Efficacy of medications, migraine	ASA 1 g = 0.39 [30] Sumatriptan 50 mg = 0.35 [22] Amitriptyline 100 mg daily = 0.44 [23]
Efficacy of medications, TTH	ASA 1 g = 0.75 (expert opinion) Paracetamol 1 g = 0.59 [31] Amitriptyline 100 mg daily = 0.3 (expert opinion)
Efficacy of withdrawal, MOH	Efficacy = reverted to migraine 85%*2/3 + reverted to TTH 85%*1/3 + unchanged but off medications 15% (expert opinion)
Uptake	
Uptake of medications, migraine	ASA 1 g = 0.635 (expert opinion) Sumatriptan 50 mg = 0.005 (expert opinion) Amitriptyline 100 mg = 0.007 (expert opinion)
Uptake of medications, TTH	Analgesic ASA 1 g = 0.1 (expert opinion) Paracetamol 1 g = 0.456 (expert opinion) Amitriptyline 100 mg = 0 (expert opinion)
Treatment efficacy (weighted by medications uptake)	
Efficacy*uptake calculations	One medication = [proportion with effect] * [effect] * [uptake]; Combination of two medications = [(proportion with effect)_medication A_ * (effect)_medication A_ * (uptake)_medication A_] + [(proportion with effect)_medication B_ * (effect)_medication B_ * (uptake)_medication B_]
Healthy life years (HLYs)	
HLYs untreated = DW * proportion of time with headache (= number/year * duration in years); HLYs treated = HLYs untreated - HLY gained from treatment; HLYs gained = HLYs untreated * efficacy; Total HLYs gained per person = sum of (gains from each treatment * probability of having each treatment) HLYs gained across the population = HLY gained per person affected * prevalence.	
HLYs MOH treated = unchanged in 15% + migraine treated HLYs in 2/3*42.5% + TTH treated HLYs in 1/3*42.5%.	

We estimated effect per person treated per time period T years as follows:

- YLDs_untreated_ = T * {(pTIS_untreated_ * ictal DW) [+ (1 – [pTIS_untreated_ * interictal DW]) in high-frequency cases]};
- YLDs_treated_ = T * {(pTIS_treated_ * ictal DW) [+ (1 – [pTIS_treated_ * interictal DW]) in high-frequency cases]};
- HLYs gained = YLDs_untreated_ – YLDs_treated_.

In the case of MOH, HLYs gained were offset according to the assumption that treatment success implied reversion to migraine (2/3) or TTH (1/3), with HLYs lost in accordance with these disorders treated.

HLYs gained per person under a particular treatment plan were equal to the sum of the gains from each treatment multiplied by the probability of having each treatment. HLYs gained in the population were equal to HLYs gained per person multiplied by the prevalence of the disorder. Assumptions adopted when calculating the health effects are summarised in Table 5.

## Results

Here we set out results for the three countries in terms of headache-related costs (including use of health-care resources and lost productivity) and health outcomes (HLYs) attached to each alternative (current vs target care) only to demonstrate how the models worked. Analyses of the differences in costs and health outcomes between alternatives and the incremental cost-effectiveness ratios are presented elsewhere [15].

### Decision-analytical models: treatment management plan and selection of interventions

Figures 2, 3 and 4 represent the separate decision analytical models developed and applied to the three country settings. The complete lists of medications, corresponding uptake estimates and assumptions made according to the different management plans (current and target alternatives) for the three headache types are set out in Tables 1, 2 and 3.

### Economic outcomes

Tables 6, 7 and 8 report the economic outcomes attached to different treatment plans for each headache type and each country. A breakdown of the different headache costs is reported for types of medication, types of consultation, examinations and lost productivity. The same calculations are repeated for each alternative (current vs target care) and for the differences between these. For MOH, summary numbers for health-care costs and lost productivity are provided (Table 8), whereas we refer to Tables 6 and 7 (target care scenario) for costs incurred by reversion to migraine or TTH. Two separate sets of data are provided for one-year and five-year time frames.

**Table 6:** Migraine costs (population estimates)

	Luxembourg *N* = 124,713		Russia *N* = 18,122,512		Spain *N* = 10,772,263	
	Cost (euros) 1-year estimate	Cost (euros) 5-year estimate	Cost (euros) 1-year estimate	Cost (euros) 5-year estimate	Cost (euros) 1-year estimate	Cost (euros) 5-year estimate
CURRENT CARE: NO CONSUMER EDUCATION OR HEALTH-CARE PROFESSIONAL TRAINING						
Primary analysis						
Medications						
acute non-specific ASA 1 g	7568	35,364	855,296	3,996,867	508,399	2,375,791
acute-specific sumatriptan 50 mg	3635	16,986	1,207,420	5,642,371	251,626	1,175,870
prophylaxis amitriptyline 100 mg/day	695	3249	195,304	912,670	20,816	97,276
Total medications	11,898	55,599	2,258,020	10,551,908	780,841	3,648,937
Health-care provision						
GP visits	1,868,796	6,153,787	133,597,931	493,496,811	185,508,849	725,403,433
specialist visits	708,809	708,809	126,073,602	126,073,602	125,445,228	125,445,229
MRI	361,613	361,613	77,008,823	77,008,823	73,240,116	73,240,116
Total health-care provision	2,939,218	7,224,209	336,680,356	696,579,236	384,194,193	924,088,778
Secondary analyses						
Total lost productivity	120,885,706	564,908,477	2,045,525,786	9,558,904,021	4,136,426,354	19,329,847,995
Sensitivity analysis: disability accounts for 20% lost productivity	24,177,141	112,981,695	409,105,157	1,911,780,804	827,285,271	3,865,969,599
TARGET CARE: WITH CONSUMER EDUCATION AND HEALTH-CARE PROFESSIONAL TRAINING						
Primary analysis						
Medications						
acute non-specific ASA 1 g	16,564	77,405	1,498,933	7,004,632	370,931	1,733,392
acute-specific sumatriptan 50 mg	153,854	718,974	50,269,478	234,913,251	10,313,113	48,193,995
prophylaxis amitriptyline 100 mg/day	30,050	140,425	7,857,564	36,719,020	899,802	4,204,845
Total medications	200,468	936,804	59,625,975	278,636,903	11,583,846	54,132,232
Health-care provision						
GP visits	4,357,297	12,893,134	373,410,366	1,104,912,154	399,420,967	1,181,876,886
specialist visits	611,042	611,042	74,160,942	74,160,942	46,119,569	46,119,569
MRI	62,347	62,347	9,059,861	9,059,861	5,385,303	5,385,303
Total health-care provision	5,030,686	13,566,523	456,631,169	1,188,132,957	450,925,839	1,233,381,758
Secondary analyses						
Total lost productivity	5,431,622	373,419,228	1,296,237,360	6,057,419,858	2,621,228,447	12,249,208,158
Sensitivity analysis: disability accounts for 20% lost productivity	1,086,324	74,683,846	259,247,472	1,211,483,972	524,245,689	2,449,841,632

**Table 7:** TTH costs (population estimates)

	Luxembourg *N* = 127,501		Russia *N* = 26,679,239		Spain *N* = 7,850,265	
	Cost (euros) 1-year estimate	Cost (euros) 5-year estimate	Cost (euros) 1-year estimate	Cost (euros) 5-year estimate	Cost (euros) 1-year estimate	Cost (euros) 5-year estimate
CURRENT CARE: NO CONSUMER EDUCATION OR HEALTH-CARE PROFESSIONAL TRAINING						
Primary analysis						
Medications						
acute ASA 1 g or paracetamol 1 g	21,135	98,765	3,439,657	16,073,791	1,012,106	4,729,652.69
prophylaxis amitriptyline 100 mg/day	–	–	–	–	–	–
Total medications	21,135	98,765	3,439,657	16,073,791	1,012,106	4,729,652.69
Health-care provision						
GP visits	710,354	1,658,990	87,659,562	204,723,838	46,415,831	108,401,488
specialist visits	324,834	324,834	56,770,353	56,770,352	17,476,479	17,476,480
MRI	66,288	66,288	13,870,685	13,870,685	4,081,397	4,081,397
Health-care provision	1,101,476	2,050,112	158,300,600	275,364,875	67,973,707	129,959,365
Secondary analyses						
Total lost productivity	46,505,330	217,323,093	1,133,152,091	5,295,309,478	1,134,307,720	5,300,709,824
Sensitivity analysis: disability accounts for 20% lost productivity	9,301,066	43,464,618	226,630,418	1,059,061,896	226,861,544	1,060,141,965
TARGET CARE: WITH CONSUMER EDUCATION AND HEALTH-CARE PROFESSIONAL TRAINING						
Primary analysis						
Medications						
acute ASA 1 g or paracetamol 1 g	31,184	145,724	4,063,501	18,989,061	497,776	2,326,148
prophylaxis amitriptyline 100 mg/day	3122	14,590	1,175,568	5,493,524	66,639	311,410
Total medications	34,306	160,314	5,239,069	24,482,585	564,415	2,637,558
Health-care provision						
GP visits	222,734	222,734	13,742,996	13,742,996	14,553,862	14,553,862
specialist visits	281,115	281,115	49,129,551	49,129,551	15,124,295	15,124,295
MRI	31,870	31,870	6,668,783	6,668,783	1,962,264	1,962,264
Total health-care provision	535,719	535,719	69,541,330	69,541,330	31,640,421	31,640,421
Secondary analyses						
Total lost productivity	45,491,467	146,579,889	764,287,428	3,571,575,691	765,066,876	3,575,218,111
Sensitivity analysis: disability accounts for 20% lost productivity	20,397,967	29,315,978	152,857,486	714,315,138	153,013,375	715,043,622

**Table 8:** MOH costs (population estimates)

	Luxembourg *N* = 14,378		Russia *N* = 7,193,081		Spain *N* = 2,128,185	
	Cost (euros) 1-year estimate	Cost (euros) 5-year estimate	Cost (euros) 1-year estimate	Cost (euros) 5-year estimate	Cost (euros) 1-year estimate	Cost (euros) 5-year estimate
CURRENT CARE: NO CONSUMER EDUCATION OR HEALTH-CARE PROFESSIONAL TRAINING						
Primary analysis						
Total health-care costs	571,570	2,670,992	168,632,151	788,031,399	89,782,290	419,559,753
Secondary analyses						
Total lost productivity	64,622,955	301,988,186	3,764,597,029	17,592,260,104	3,789,174,825	17,707,114,095
Sensitivity analysis: disability accounts for 20% lost productivity	12,924,591	60,397,637	752,919,406	3,518,452,021	757,834,965	541,422,818.96
TARGET CARE: WITH CONSUMER EDUCATION AND HEALTH-CARE PROFESSIONAL TRAINING						
Primary analysis						
Total health-care costs	266,932	2,670,992	86,693,089	405,123,672	40,755,568	190,453,998
Secondary analyses						
Total lost productivity	9,011,897	42,113,311	520,108,800	2,430,509,620	523,504,417	2,446,377,605
Sensitivity analysis: disability accounts for 20% lost productivity	1,802,379	8,422,662	104,021,760	486,101,924	104,700,883	489,275,521

For example, for the estimated 18,122,512 Russians with migraine (Table 6):

- current care required 303,241,487 euros invested in health care over 1 year, whereas
- target care (with consumer education and health-care provider training) would require 575,883,120 euros invested in health care over 1 year.

### Health outcomes

Table 4 reports calculated headache-attributed disabilities at individual level. Tables 9, 10 and 11 report annual HLYs potentially gained by each element of the proposed treatment plan for each headache type in each country. The same calculations are again repeated for each alternative (current vs target care) and for the differences between these. The population-level effect on health of the intervention strategies for target care, through reduced pTIS (achieved through SHR and/or reduced attack frequency), is quite substantial.

For example, for all Russians with migraine:

- current care gained an estimated 158,406 HLYs, whereas
- target care (with consumer education and health care professional training) would secure 322,115 HLYs gained (163,709 more than current care; Table 9).

**Table 9:** Healthy Life Years (HLYs) potentially gained in 1 year by each element of the proposed intervention (migraine)

	Efficacy * uptake	Healthy Life Years per capita			Luxembourg *N* = 124,713		Russia *N* = 18,122,512		Spain *N* = 10,772,263	
		Not treated	Treated	Gained	Affected individuals under treatment (n)	HLYs gained across population	Affected individuals under treatment (n)	HLYs gained across population	Affected individuals under treatment (n)	HLYs gained across population
CURRENT CARE: NO CONSUMER EDUCATION OR HEALTH-CARE PROFESSIONAL TRAINING										
A. Acute management (non-specific drugs) ASA 1 g	0.2146	0.0399	0.0313	0.0086	79,193	0.0026	11,507,795	0.0015	6,840,387	0.0030
B. Acute management (specific drugs) sumatriptan 50 mg	0.0015	0.0399	0.0398	0.0001	624	1.83579E-05	90,613	1.08171E-05	53,861	0.0000
C. Acute stepped-care management ASA 1 g + sumatriptan 50 mg	0	0.0399	0.0399	0	0	0	0	0	0	0
D. Prophylaxis + acute management amitriptyline 100 mg/day + ASA 1 g + sumatriptan 50 mg	0.0031	0.0399	0.0397	0.0001	873	3.72807E-05	126,858	2.19671E-05	75,406	0.00004
Total					80,690	0.002656	11,725,266	0.001533	6,969,654	0.00304
HLYs FOR OVERALL POPULATION					**1090**		**158,406**		**94,159**	
TARGET CARE: WITH CONSUMER EDUCATION AND HEALTH-CARE PROFESSIONAL TRAINING										
A. Acute management (non-specific drugs) ASA 1 g	0.1386	0.0399	0.0343	0.0055	51,132	0.0017	7,430,230	0.0010	4,416,628	0.0020
B. Acute management (specific drugs) sumatriptan 50 mg	0	0.0399	0.0399	0	0	0		0		0
C. Acute stepped-care management ASA 1 g + sumatriptan 50 mg	0.1052	0.0399	0.0357	0.0042	153,854	0.0013	50,269,478	0.0008	10,313,113	0.0015
D. Prophylaxis + acute management amitriptyline 100 mg/day + ASA 1 g + sumatriptan 50 mg	0.2020	0.0399	0.0318	0.0081	30,679	0.0024	4,458,138	0.0014	2,649,977	0.0029
Total					235,666	0.0054	62,157,845	0.0032	17,379,718	0.0063
HLYs FOR OVERALL POPULATION					**2217**		**322,115**		**191,470**

**Table 10:** Healthy Life Years (HLYs) potentially gained in 1 year by each element of the proposed intervention (TTH)

	Efficacy * uptake	Healthy Life Years per capita			Luxembourg *N* = 124,713		Russia *N* = 18,122,512	Spain *N* = 10,772,263		
		Not treated	Treated	Gained	Affected individuals under treatment (n)	HLYs gained across population	Affected individuals under treatment (n)	HLYs gained across population	Affected individuals under treatment (n)	HLYs gained across population
CURRENT CARE: NO CONSUMER EDUCATION OR HEALTH-CARE PROFESSIONAL TRAINING										
Acute management ASA 1 g or paracetamol 1 g	0.3340	0.0013	0.0009	0.0004	70,890	0.0001	14,833,657	0.00012	4,364,748	0.00011
Prophylaxis + acute management amitriptyline 100 mg/day + ASA 1 g or paracetamol 1 g	0.3340	0.0013	0.0009	0.0004	0	0.0001	0	0.00012	0	0.00011
Total					70,890	0.0002	14,833,657	0.0026	4,364,748	0.00022
HLYs FOR OVERALL POPULATION					**112**		**23,398**		**6885**	
TARGET CARE: WITH CONSUMER EDUCATION AND HEALTH-CARE PROFESSIONAL TRAINING										
Acute management ASA 1 g or paracetamol 1 g	0.4847	0.0013	0.0007	0.0006	96,263	0.0002	20,142,825	0.00017	5,926,950	0.00016
Prophylaxis + acute management amitriptyline 100 mg/day + ASA 1 g or paracetamol 1 g	0.4886	0.0013	0.0007	0.0006	3188	0.0002	666,981	0.00017	196,257	0.00017
Total					99,451	0.0004	20,809,806	0.0026	6,123,207	0.00033
HLYs FOR OVERALL POPULATION					**163**		**34,090**		**10,031**	

## Discussion

This study presents the first headache-type-specific analytical models for comparing the effectiveness and cost-effectiveness of implemented structured headache services across European Region country settings. The models linked direct costs (resources sunk into health-care provision) and indirect costs (lost work productivity) with health outcomes (in terms of HLYs). While the literature does provide a framework to assess population-level cost-effectiveness of evidence-based migraine *treatments* in low- and middle-income countries [19], data are very scarce on costs and effects of introducing headache *services* enhancing treatment delivery through a better-defined care pathway [9]. The methodology was successful, bringing together observed data for current care and estimates for target care. The flexibility of the models allowed measurements of the benefits, in people with different headache types, of care improvements achieved through implementing structured services in different countries.

The countries included – Luxembourg, Russia and Spain – were diverse in terms of geographical location, population size, level of income and organisation of their health-care systems. For example, Luxembourg was chosen because their health-care system is perceived as one of the best in Europe [36]. It has a high standard of state-funded health care covering every citizen, each having the right to choose their doctor, specialist and hospital. In Russia, although the health service is free to all, a complex compulsory medical insurance system coupled with low wages for doctors and nurses means that demands for out-of-pocket payments remain a pervasive and discouraging problem. Lastly, Spain offers free, universal health care to anyone resident, but the system is decentralised across the country’s 17 autonomous regions, so that quality of care, and access to specialist procedures or units, vary across regions.

The population and costing models rest upon a series of best estimates, including the expected patterns of resource use and intervention efficacy. Data to support these in each of the three countries were sourced from population-based studies in Russia [17, 18] and the Eurolight project for Luxembourg and Spain [16]. Unfortunately, in Eurolight, participation proportions were suboptimal and samples might not be truly representative [36]. Participants were not asked about formulations of acute medicines, and the numbers of doses were estimated conservatively. For preventative medicines, it was assumed that recommended doses were used [8, 19].

In addition, effectiveness data were drawn from published controlled trials, which did not always include the countries in question. All findings might also be sensitive to assumptions made in the costing model, and to possible variations in the national statistics applied (see Table 4).

**Table 11:** Healthy Life Years (HLYs) potentially gained in 1 year by each element of the proposed intervention (MOH)

	Healthy Life Years per capita			Luxembourg *N* = 124,713		Russia *N* = 18,122,512		Spain *N* = 10,772,263	
	Not treated	Treated	Gained	Affected individuals under treatment (n)	HLYs gained across population	Affected individuals under treatment (n)	HLYs gained across population	Affected individuals under treatment (n)	HLYs gained across population
CURRENT CARE: NO CONSUMER EDUCATION AND HEALTH CARE PROFESSIONAL TRAINING									
No treatment coverage	0.1671	0	0	0	0	0	0	0	0
HLYs FOR OVERALL POPULATION					**0**		**0**		**0**
TARGET CARE: WITH CONSUMER EDUCATION AND HEALTH CARE PROFESSIONAL TRAINING									
Treatment coverage	0.1671	0.2318	0.0647	6110	0.0046	3,057,059	0.0046	904,478	0.0046
HLYs FOR OVERALL POPULATION				**776**		**388,112**		**114,829**	

Even though the indirect costs of migraine and MOH dominate the direct costs, productivity gains and lost-time costs were not taken into consideration in our main analyses because no internationally agreed approach is yet available to measure these satisfactorily [7, 11]. However, in our sensitivity analyses, we used the human capital approach as the most common method for estimating the economic value of employee productivity, assuming that it is equal to gross earnings [21]. This allowed us to re-run the models from the broader societal perspective, covering both health-care provider costs and those due to lost productivity.

A major difficulty lay in the relationship between headache-attributed disability, estimated from DWs generated in GBD2015, and headache-attributed lost work productivity. A strong correlation was intuitively expected. In our baseline scenario, we assumed that lost productivity was fully explained by headache-attributed disability: *ie*, reductions in disability would bring commensurate reductions in lost productivity. This may not be so at population level because, as mentioned earlier, people are variably influenced by a number of extraneous and sometimes random factors [24]. The sensitivity analyses showed that varying the proportion of lost productivity recovered had a major impact on economic estimates. This was expected, because predicted savings in work productivity greatly exceeded the investments in health care estimated to be needed to achieve these savings. Nevertheless, in a conservative scenario, where we assumed that remedying disability would recover only 20% of the lost productivity attributed to it, the intervention remained cost-effective in all models and cost-saving in Luxembourg. Furthermore, at *individual* level (relevant in the context of treatment), the extraneous factors are mostly constant, meaning a simpler and closer relationship was likely (Hallie Thomas, Simple Futarmal Kothari, Andreas Husøy, Rigmor Hølland Jensen, Zaza Katsarava, Michela Tinell and Timothy J Steiner. The relationship between headache-attributed disability and lost productivity. 2. Empirical evidence from population-based studies in six disparate countries forthcoming).

## Conclusion

Despite these limitations, the study delivered robust models, with detailed results presented in the next paper in this series [15]. The models should greatly assist local health-policy makers, across Europe and very probably elsewhere, in allocating fixed health budgets between interventions to maximise health in society. Health-care systems vary widely even within the European Region, and certainly outside it, but the analytical models should be applicable to any that adopt and fully implement the services model [9]. Widely different costs (such as input costs and income levels) may of course lead to different analytical outcomes.

## Data Availability

All data generated or analysed during this study are included in this published article.

## Appendix

**Table 12:** Unit costs

	Luxembourg Cost per year per capita (euro) 2020	Russia Cost per year per capita (euro) 2020	Spain Cost per year per capita (euro) 2020	References
MEDICINES				[5, 17, 19]. Costs actualised to 2020 values using appropriate consumer price index [27]
A. Acute management (non-specific drugs)				
ASA 1 g	0.10	0.07	0.07	
Paracetamol 1 g	0.297	0.23	0.23	
With consumer education	0.33	0.21	0.08	
B. Acute management (specific drugs)				
Sumatriptan 50 mg	6.06	13.85	4.86	
With consumer education and provider training	6.07	14.09	4.87	
C. Acute stepped-care management				
ASA 1 g + sumatriptan 50 mg	6.16	17.35	4.94	
With consumer education and provider training	7.82	17.59	6.07	
D. Prophylaxis + acute management				
Amitriptyline 100 mg + ASA 1 g + sumatriptan 50 mg	0.83	1.60	0.29	
With consumer education and provider training	1.02	1.83	0.35	
VISITS				
One GP visit	38.76	22.86	41.13	[25]
One specialist visit	48.92	40.85	42.74	[25]
EXAMINATION				
One MRI	49.91	49.91	49.91	[7]
PRODUCTIVITY				
Daily wages	205.01	23.87	81.21	[27]

## Abbreviations

ASA: Acetylsalicylic acid
D: Expected attack duration in hours
DWs: Disability weights
EBC: European Brain Council
F: Frequency
GBD: Global Burden of Disease
HLY: Healthy Life Year
HLDs: Hours lived with disability
ICERs: Incremental cost-effectiveness ratios
MOH: Medication-overuse headache
pTIS: Proportion of time in ictal state
pF_treated_: Proportion treated
SHR: Sustained headache relief
TTH: Tension-type headache
U_cc_: Uptake of each treatment in current care
U_tc_: Uptake of each treatment in target care
VoT: Value of Treatment
WHO: World Health Organization
YLDs: Years lived with disability
